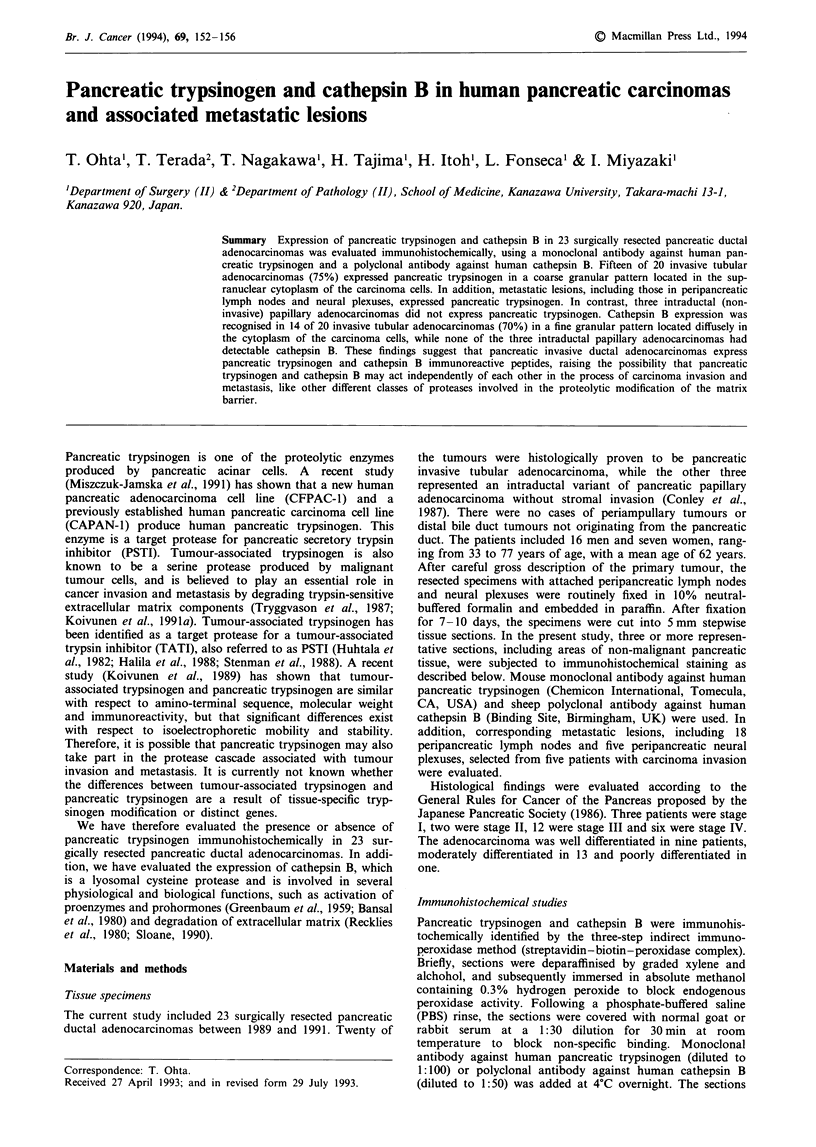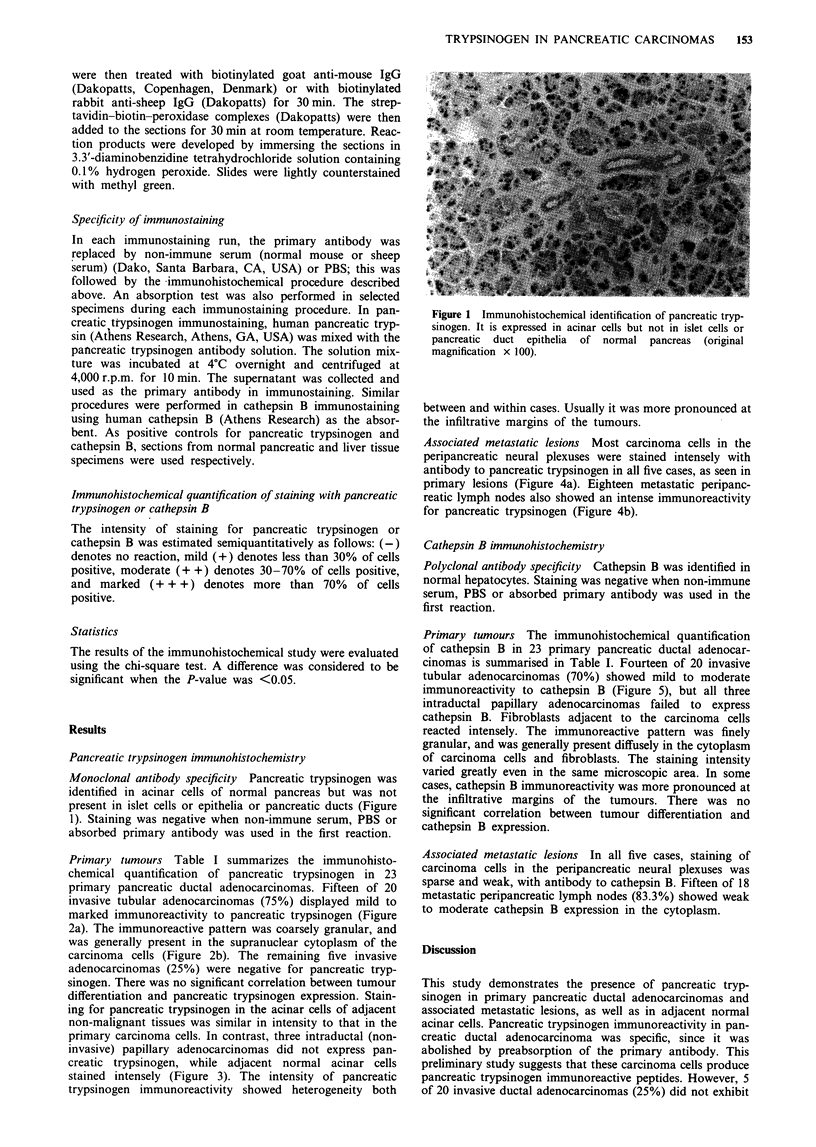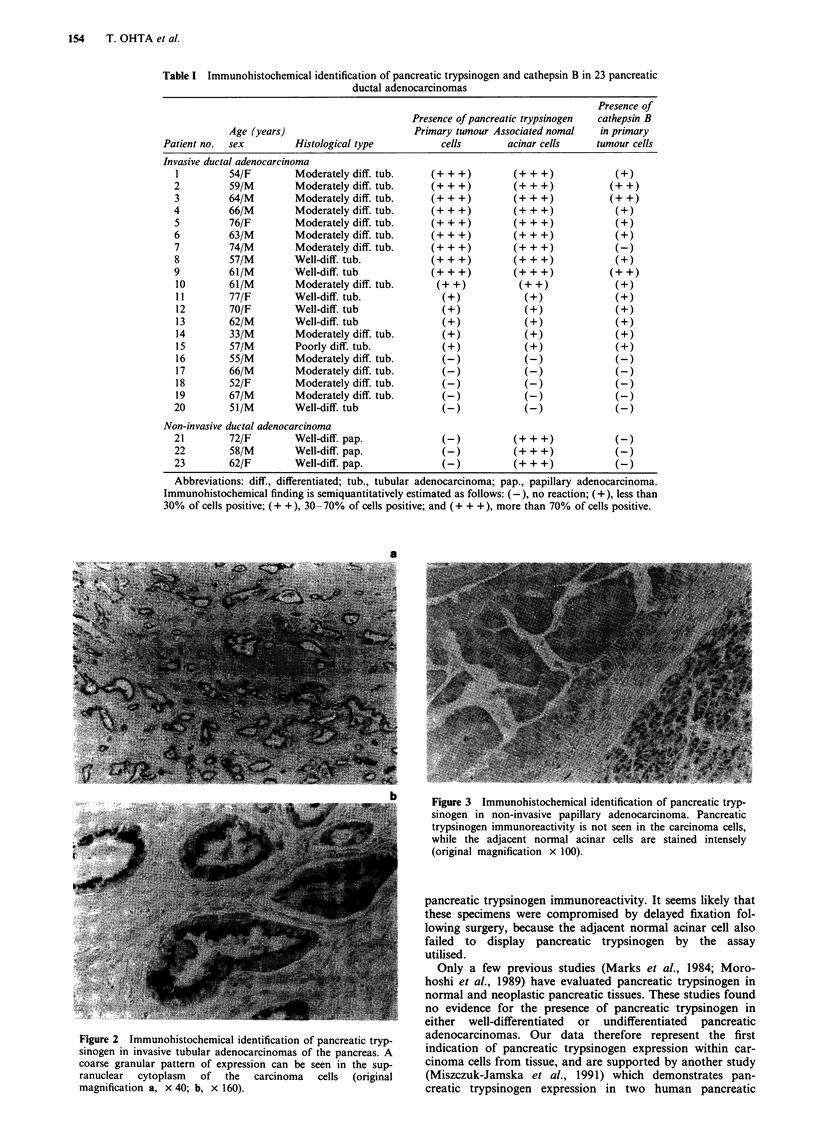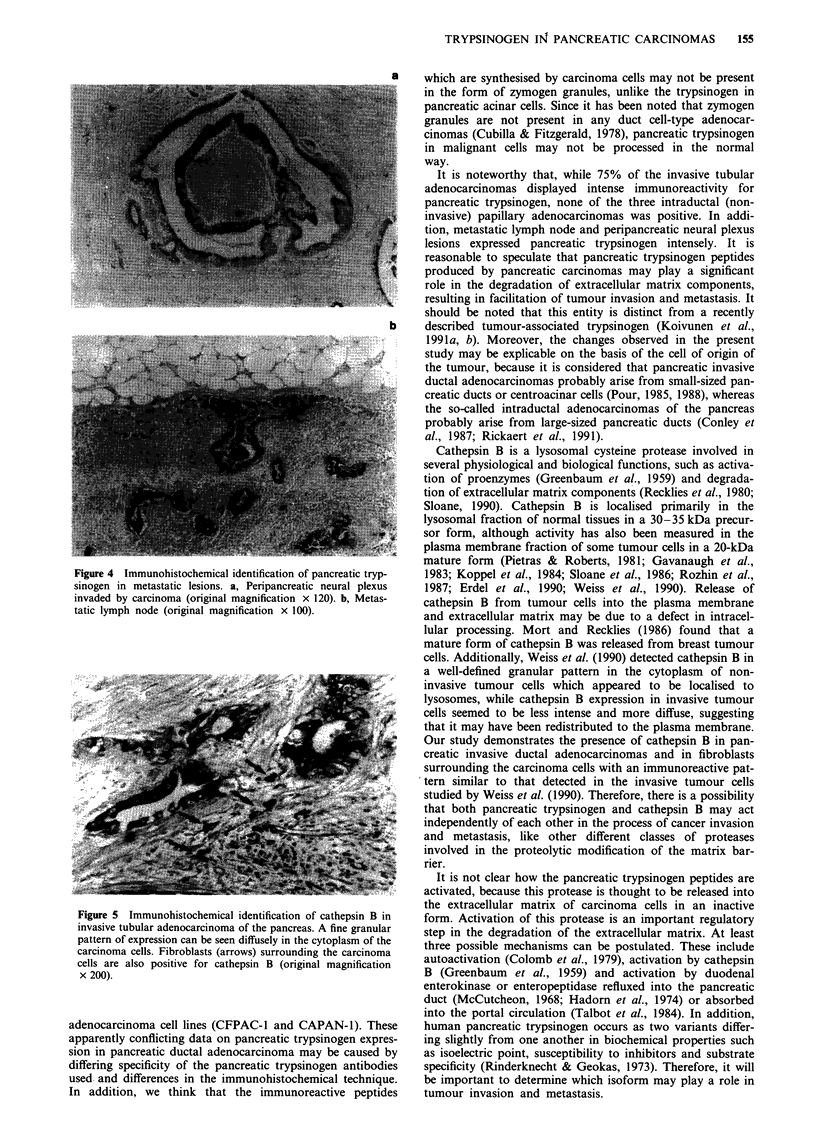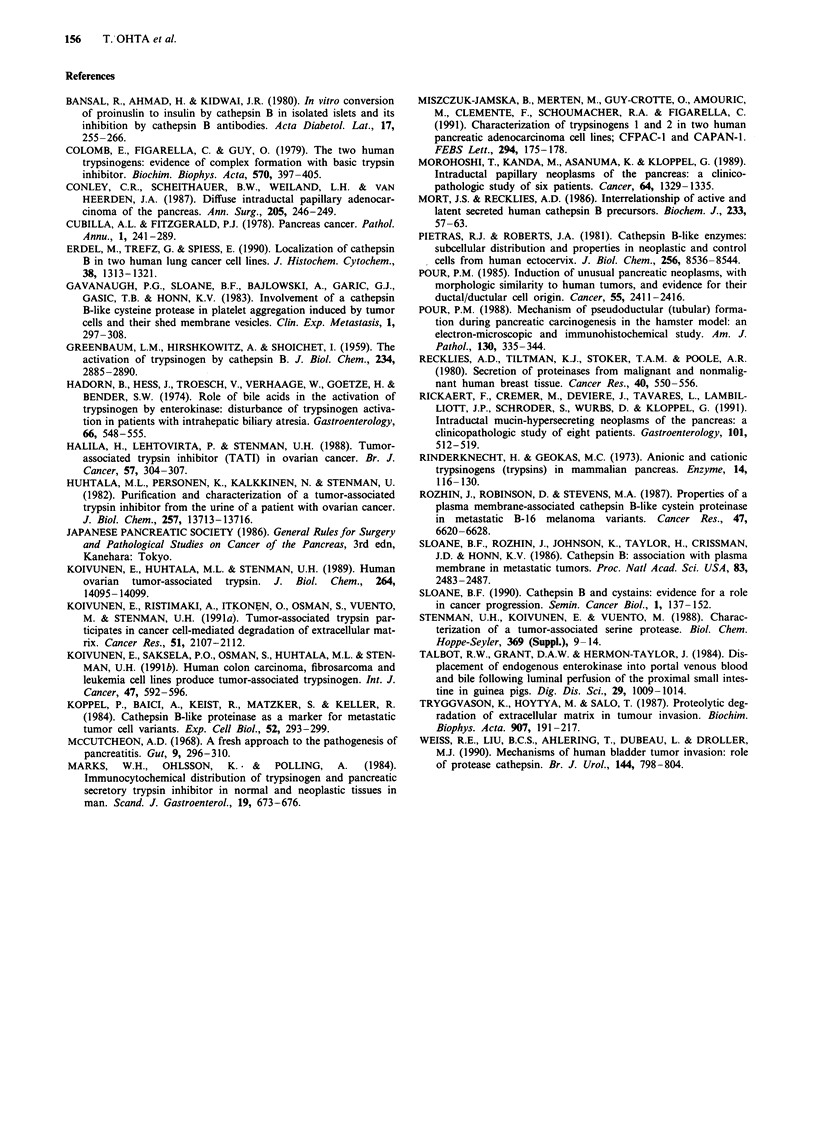# Pancreatic trypsinogen and cathepsin B in human pancreatic carcinomas and associated metastatic lesions.

**DOI:** 10.1038/bjc.1994.25

**Published:** 1994-01

**Authors:** T. Ohta, T. Terada, T. Nagakawa, H. Tajima, H. Itoh, L. Fonseca, I. Miyazaki

**Affiliations:** Department of Surgery (II), School of Medicine, Kanazawa University, Japan.

## Abstract

**Images:**


Br. J. Cancer (1994), 69, 152-156 © Macmillan Press Ltd., 1994

### Pancreatic trypsinogen and cathepsin B in human pancreatic carcinomas and associated metastatic lesions

T. Ohta[1], T. Terada[2], T. Nagakawa[1], H. Tajima[1], H. Itoh[1], L. Fonseca[1] & I. Miyazaki[1]

[1]*Department of Surgery (II) &* [2]*Department of Pathology (II), School of Medicine, Kanazawa University, Takara-machi 13-1, Kanazawa 920, Japan.*

**Summary** Expression of pancreatic trypsinogen and cathepsin B in 23 surgically resected pancreatic ductal adenocarcinomas was evaluated immunohistochemically, using a monoclonal antibody against human pancreatic trypsinogen and a polyclonal antibody against human cathepsin B. Fifteen of 20 invasive tubular adenocarcinomas (75%) expressed pancreatic trypsinogen in a coarse granular pattern located in the supranuclear cytoplasm of the carcinoma cells. In addition, metastatic lesions, including those in peripancreatic lymph nodes and neural plexuses, expressed pancreatic trypsinogen. In contrast, three intraductal (non-invasive) papillary adenocarcinomas did not express pancreatic trypsinogen. Cathepsin B expression was recognised in 14 of 20 invasive tubular adenocarcinomas (70%) in a fine granular pattern located diffusely in the cytoplasm of the carcinoma cells, while none of the three intraductal papillary adenocarcinomas had detectable cathepsin B. These findings suggest that pancreatic invasive ductal adenocarcinomas express pancreatic trypsinogen and cathepsin B immunoreactive peptides, raising the possibility that pancreatic trypsinogen and cathepsin B may act independently of each other in the process of carcinoma invasion and metastasis, like other different classes of proteases involved in the proteolytic modification of the matrix barrier.

Pancreatic trypsinogen is one of the proteolytic enzymes produced by pancreatic acinar cells. A recent study (Miszczuk-Jamska *et al.*, 1991) has shown that a new human pancreatic adenocarcinoma cell line (CFPAC-1) and a previously established human pancreatic carcinoma cell line (CAPAN-1) produce human pancreatic trypsinogen. This enzyme is a target protease for pancreatic secretory trypsin inhibitor (PSTI). Tumour-associated trypsinogen is also known to be a serine protease produced by malignant tumour cells, and is believed to play an essential role in cancer invasion and metastasis by degrading trypsin-sensitive extracellular matrix components (Tryggvason *et al.*, 1987; Koivunen *et al.*, 1991*a*). Tumour-associated trypsinogen has been identified as a target protease for a tumour-associated trypsin inhibitor (TATI), also referred to as PSTI (Huhtala *et al.*, 1982; Halila *et al.*, 1988; Stenman *et al.*, 1988). A recent study (Koivunen *et al.*, 1989) has shown that tumour-associated trypsinogen and pancreatic trypsinogen are similar with respect to amino-terminal sequence, molecular weight and immunoreactivity, but that significant differences exist with respect to isoelectrophoretic mobility and stability. Therefore, it is possible that pancreatic trypsinogen may also take part in the protease cascade associated with tumour invasion and metastasis. It is currently not known whether the differences between tumour-associated trypsinogen and pancreatic trypsinogen are a result of tissue-specific trypsinogen modification or distinct genes.

We have therefore evaluated the presence or absence of pancreatic trypsinogen immunohistochemically in 23 surgically resected pancreatic ductal adenocarcinomas. In addition, we have evaluated the expression of cathepsin B, which is a lyosomal cysteine protease and is involved in several physiological and biological functions, such as activation of proenzymes and prohormones (Greenbaum *et al.*, 1959; Bansal *et al.*, 1980) and degradation of extracellular matrix (Recklies *et al.*, 1980; Sloane, 1990).

### Materials and methods

### *Tissue specimens*

The current study included 23 surgically resected pancreatic ductal adenocarcinomas between 1989 and 1991. Twenty of the tumours were histologically proven to be pancreatic invasive tubular adenocarcinoma, while the other three represented an intraductal variant of pancreatic papillary adenocarcinoma without stromal invasion (Conley *et al.*, 1987). There were no cases of periampullary tumours or distal bile duct tumours not originating from the pancreatic duct. The patients included 16 men and seven women, ranging from 33 to 77 years of age, with a mean age of 62 years. After careful gross description of the primary tumour, the resected specimens with attached peripancreatic lymph nodes and neural plexuses were routinely fixed in 10% neutral-buffered formalin and embedded in paraffin. After fixation for 7-10 days, the specimens were cut into 5 mm stepwise tissue sections. In the present study, three or more representative sections, including areas of non-malignant pancreatic tissue, were subjected to immunohistochemical staining as described below. Mouse monoclonal antibody against human pancreatic trypsinogen (Chemicon International, Tomecula, CA, USA) and sheep polyclonal antibody against human cathepsin B (Binding Site, Birmingham, UK) were used. In addition, corresponding metastatic lesions, including 18 peripancreatic lymph nodes and five peripancreatic neural plexuses, selected from five patients with carcinoma invasion were evaluated.

Histological findings were evaluated according to the General Rules for Cancer of the Pancreas proposed by the Japanese Pancreatic Society (1986). Three patients were stage I, two were stage II, 12 were stage III and six were stage IV. The adenocarcinoma was well differentiated in nine patients, moderately differentiated in 13 and poorly differentiated in one.

### *Immunohistochemical studies*

Pancreatic trypsinogen and cathepsin B were immunohistochemically identified by the three-step indirect immunoperoxidase method (streptavidin-biotin-peroxidase complex). Briefly, sections were deparaffinised by graded xylene and alchohol, and subsequently immersed in absolute methanol containing 0.3% hydrogen peroxide to block endogenous peroxidase activity. Following a phosphate-buffered saline (PBS) rinse, the sections were covered with normal goat or rabbit serum at a 1:30 dilution for 30 min at room temperature to block non-specific binding. Monoclonal antibody against human pancreatic trypsinogen (diluted to 1:100) or polyclonal antibody against human cathepsin B (diluted to 1:50) was added at 4°C overnight. The sections

Correspondence: T. Ohta.
Received 27 April 1993; and in revised form 29 July 1993.

were then treated with biotinylated goat anti-mouse IgG (Dakopatts, Copenhagen, Denmark) or with biotinylated rabbit anti-sheep IgG (Dakopatts) for 30 min. The streptavidin–biotin–peroxidase complexes (Dakopatts) were then added to the sections for 30 min at room temperature. Reaction products were developed by immersing the sections in 3.3′-diaminobenzidine tetrahydrochloride solution containing 0.1% hydrogen peroxide. Slides were lightly counterstained with methyl green.

*Specificity of immunostaining*

In each immunostaining run, the primary antibody was replaced by non-immune serum (normal mouse or sheep serum) (Dako, Santa Barbara, CA, USA) or PBS; this was followed by the immunohistochemical procedure described above. An absorption test was also performed in selected specimens during each immunostaining procedure. In pancreatic trypsinogen immunostaining, human pancreatic trypsin (Athens Research, Athens, GA, USA) was mixed with the pancreatic trypsinogen antibody solution. The solution mixture was incubated at 4°C overnight and centrifuged at 4,000 r.p.m. for 10 min. The supernatant was collected and used as the primary antibody in immunostaining. Similar procedures were performed in cathepsin B immunostaining using human cathepsin B (Athens Research) as the absorbent. As positive controls for pancreatic trypsinogen and cathepsin B, sections from normal pancreatic and liver tissue specimens were used respectively.

*Immunohistochemical quantification of staining with pancreatic trypsinogen or cathepsin B*

The intensity of staining for pancreatic trypsinogen or cathepsin B was estimated semiquantitatively as follows: (−) denotes no reaction, mild (+) denotes less than 30% of cells positive, moderate (+ +) denotes 30–70% of cells positive, and marked (+ + +) denotes more than 70% of cells positive.

*Statistics*

The results of the immunohistochemical study were evaluated using the chi-square test. A difference was considered to be significant when the $P$-value was $<0.05$.

### Results

*Pancreatic trypsinogen immunohistochemistry*

*Monoclonal antibody specificity* Pancreatic trypsinogen was identified in acinar cells of normal pancreas but was not present in islet cells or epithelia or pancreatic ducts (Figure 1). Staining was negative when non-immune serum, PBS or absorbed primary antibody was used in the first reaction.

*Primary tumours* Table I summarizes the immunohistochemical quantification of pancreatic trypsinogen in 23 primary pancreatic ductal adenocarcinomas. Fifteen of 20 invasive tubular adenocarcinomas (75%) displayed mild to marked immunoreactivity to pancreatic trypsinogen (Figure 2a). The immunoreactive pattern was coarsely granular, and was generally present in the supranuclear cytoplasm of the carcinoma cells (Figure 2b). The remaining five invasive adenocarcinomas (25%) were negative for pancreatic trypsinogen. There was no significant correlation between tumour differentiation and pancreatic trypsinogen expression. Staining for pancreatic trypsinogen in the acinar cells of adjacent non-malignant tissues was similar in intensity to that in the primary carcinoma cells. In contrast, three intraductal (non-invasive) papillary adenocarcinomas did not express pancreatic trypsinogen, while adjacent normal acinar cells stained intensely (Figure 3). The intensity of pancreatic trypsinogen immunoreactivity showed heterogeneity both

**Figure 1** Immunohistochemical identification of pancreatic trypsinogen. It is expressed in acinar cells but not in islet cells or pancreatic duct epithelia of normal pancreas (original magnification × 100).

between and within cases. Usually it was more pronounced at the infiltrative margins of the tumours.

*Associated metastatic lesions* Most carcinoma cells in the peripancreatic neural plexuses were stained intensely with antibody to pancreatic trypsinogen in all five cases, as seen in primary lesions (Figure 4a). Eighteen metastatic peripancreatic lymph nodes also showed an intense immunoreactivity for pancreatic trypsinogen (Figure 4b).

*Cathepsin B immunohistochemistry*

*Polyclonal antibody specificity* Cathepsin B was identified in normal hepatocytes. Staining was negative when non-immune serum, PBS or absorbed primary antibody was used in the first reaction.

*Primary tumours* The immunohistochemical quantification of cathepsin B in 23 primary pancreatic ductal adenocarcinomas is summarised in Table I. Fourteen of 20 invasive tubular adenocarcinomas (70%) showed mild to moderate immunoreactivity to cathepsin B (Figure 5), but all three intraductal papillary adenocarcinomas failed to express cathepsin B. Fibroblasts adjacent to the carcinoma cells reacted intensely. The immunoreactive pattern was finely granular, and was generally present diffusely in the cytoplasm of carcinoma cells and fibroblasts. The staining intensity varied greatly even in the same microscopic area. In some cases, cathepsin B immunoreactivity was more pronounced at the infiltrative margins of the tumours. There was no significant correlation between tumour differentiation and cathepsin B expression.

*Associated metastatic lesions* In all five cases, staining of carcinoma cells in the peripancreatic neural plexuses was sparse and weak, with antibody to cathepsin B. Fifteen of 18 metastatic peripancreatic lymph nodes (83.3%) showed weak to moderate cathepsin B expression in the cytoplasm.

### Discussion

This study demonstrates the presence of pancreatic trypsinogen in primary pancreatic ductal adenocarcinomas and associated metastatic lesions, as well as in adjacent normal acinar cells. Pancreatic trypsinogen immunoreactivity in pancreatic ductal adenocarcinoma was specific, since it was abolished by preabsorption of the primary antibody. This preliminary study suggests that these carcinoma cells produce pancreatic trypsinogen immunoreactive peptides. However, 5 of 20 invasive ductal adenocarcinomas (25%) did not exhibit

**Table I** Immunohistochemical identification of pancreatic trypsinogen and cathepsin B in 23 pancreatic ductal adenocarcinomas

| Patient no. | Age (years) sex | Histological type | Presence of pancreatic trypsinogen Primary tumour cells | Presence of pancreatic trypsinogen Associated nomal acinar cells | Presence of cathepsin B in primary tumour cells |
|---|---|---|---|---|---|
| *Invasive ductal adenocarcinoma* | | | | | |
| 1 | 54/F | Moderately diff. tub. | (+ + +) | (+ + +) | (+) |
| 2 | 59/M | Moderately diff. tub. | (+ + +) | (+ + +) | (+ +) |
| 3 | 64/M | Moderately diff. tub. | (+ + +) | (+ + +) | (+ +) |
| 4 | 66/M | Moderately diff. tub. | (+ + +) | (+ + +) | (+) |
| 5 | 76/F | Moderately diff. tub. | (+ + +) | (+ + +) | (+) |
| 6 | 63/M | Moderately diff. tub. | (+ + +) | (+ + +) | (+) |
| 7 | 74/M | Moderately diff. tub. | (+ + +) | (+ + +) | (−) |
| 8 | 57/M | Well-diff. tub. | (+ + +) | (+ + +) | (+) |
| 9 | 61/M | Well-diff. tub | (+ + +) | (+ + +) | (+ +) |
| 10 | 61/M | Moderately diff. tub. | (+ +) | (+ +) | (+) |
| 11 | 77/F | Well-diff. tub. | (+) | (+) | (+) |
| 12 | 70/F | Well-diff. tub | (+) | (+) | (+) |
| 13 | 62/M | Well-diff. tub | (+) | (+) | (+) |
| 14 | 33/M | Moderately diff. tub. | (+) | (+) | (+) |
| 15 | 57/M | Poorly diff. tub. | (+) | (+) | (+) |
| 16 | 55/M | Moderately diff. tub. | (−) | (−) | (−) |
| 17 | 66/M | Moderately diff. tub. | (−) | (−) | (−) |
| 18 | 52/F | Moderately diff. tub. | (−) | (−) | (−) |
| 19 | 67/M | Moderately diff. tub. | (−) | (−) | (−) |
| 20 | 51/M | Well-diff. tub | (−) | (−) | (−) |
| *Non-invasive ductal adenocarcinoma* | | | | | |
| 21 | 72/F | Well-diff. pap. | (−) | (+ + +) | (−) |
| 22 | 58/M | Well-diff. pap. | (−) | (+ + +) | (−) |
| 23 | 62/F | Well-diff. pap. | (−) | (+ + +) | (−) |

Abbreviations: diff., differentiated; tub., tubular adenocarcinoma; pap., papillary adenocarcinoma. Immunohistochemical finding is semiquantitatively estimated as follows: (−), no reaction; (+), less than 30% of cells positive; (+ +), 30–70% of cells positive; and (+ + +), more than 70% of cells positive.

**Figure 2** Immunohistochemical identification of pancreatic trypsinogen in invasive tubular adenocarcinomas of the pancreas. A coarse granular pattern of expression can be seen in the supranuclear cytoplasm of the carcinoma cells (original magnification **a**, × 40; **b**, × 160).

**Figure 3** Immunohistochemical identification of pancreatic trypsinogen in non-invasive papillary adenocarcinoma. Pancreatic trypsinogen immunoreactivity is not seen in the carcinoma cells, while the adjacent normal acinar cells are stained intensely (original magnification × 100).

pancreatic trypsinogen immunoreactivity. It seems likely that these specimens were compromised by delayed fixation following surgery, because the adjacent normal acinar cell also failed to display pancreatic trypsinogen by the assay utilised.

Only a few previous studies (Marks *et al.*, 1984; Morohoshi *et al.*, 1989) have evaluated pancreatic trypsinogen in normal and neoplastic pancreatic tissues. These studies found no evidence for the presence of pancreatic trypsinogen in either well-differentiated or undifferentiated pancreatic adenocarcinomas. Our data therefore represent the first indication of pancreatic trypsinogen expression within carcinoma cells from tissue, and are supported by another study (Miszczuk-Jamska *et al.*, 1991) which demonstrates pancreatic trypsinogen expression in two human pancreatic

**Figure 4** Immunohistochemical identification of pancreatic trypsinogen in metastatic lesions. **a**, Peripancreatic neural plexus invaded by carcinoma (original magnification × 120). **b**, Metastatic lymph node (original magnification × 100).

**Figure 5** Immunohistochemical identification of cathepsin B in invasive tubular adenocarcinoma of the pancreas. A fine granular pattern of expression can be seen diffusely in the cytoplasm of the carcinoma cells. Fibroblasts (arrows) surrounding the carcinoma cells are also positive for cathepsin B (original magnification × 200).

adenocarcinoma cell lines (CFPAC-1 and CAPAN-1). These apparently conflicting data on pancreatic trypsinogen expression in pancreatic ductal adenocarcinoma may be caused by differing specificity of the pancreatic trypsinogen antibodies used and differences in the immunohistochemical technique. In addition, we think that the immunoreactive peptides which are synthesised by carcinoma cells may not be present in the form of zymogen granules, unlike the trypsinogen in pancreatic acinar cells. Since it has been noted that zymogen granules are not present in any duct cell-type adenocarcinomas (Cubilla & Fitzgerald, 1978), pancreatic trypsinogen in malignant cells may not be processed in the normal way.

It is noteworthy that, while 75% of the invasive tubular adenocarcinomas displayed intense immunoreactivity for pancreatic trypsinogen, none of the three intraductal (non-invasive) papillary adenocarcinomas was positive. In addition, metastatic lymph node and peripancreatic neural plexus lesions expressed pancreatic trypsinogen intensely. It is reasonable to speculate that pancreatic trypsinogen peptides produced by pancreatic carcinomas may play a significant role in the degradation of extracellular matrix components, resulting in facilitation of tumour invasion and metastasis. It should be noted that this entity is distinct from a recently described tumour-associated trypsinogen (Koivunen *et al.*, 1991*a*, *b*). Moreover, the changes observed in the present study may be explicable on the basis of the cell of origin of the tumour, because it is considered that pancreatic invasive ductal adenocarcinomas probably arise from small-sized pancreatic ducts or centroacinar cells (Pour, 1985, 1988), whereas the so-called intraductal adenocarcinomas of the pancreas probably arise from large-sized pancreatic ducts (Conley *et al.*, 1987; Rickaert *et al.*, 1991).

Cathepsin B is a lysosomal cysteine protease involved in several physiological and biological functions, such as activation of proenzymes (Greenbaum *et al.*, 1959) and degradation of extracellular matrix components (Recklies *et al.*, 1980; Sloane, 1990). Cathepsin B is localised primarily in the lysosomal fraction of normal tissues in a 30–35 kDa precursor form, although activity has also been measured in the plasma membrane fraction of some tumour cells in a 20-kDa mature form (Pietras & Roberts, 1981; Gavanaugh *et al.*, 1983; Koppel *et al.*, 1984; Sloane *et al.*, 1986; Rozhin *et al.*, 1987; Erdel *et al.*, 1990; Weiss *et al.*, 1990). Release of cathepsin B from tumour cells into the plasma membrane and extracellular matrix may be due to a defect in intracellular processing. Mort and Recklies (1986) found that a mature form of cathepsin B was released from breast tumour cells. Additionally, Weiss *et al.* (1990) detected cathepsin B in a well-defined granular pattern in the cytoplasm of non-invasive tumour cells which appeared to be localised to lysosomes, while cathepsin B expression in invasive tumour cells seemed to be less intense and more diffuse, suggesting that it may have been redistributed to the plasma membrane. Our study demonstrates the presence of cathepsin B in pancreatic invasive ductal adenocarcinomas and in fibroblasts surrounding the carcinoma cells with an immunoreactive pattern similar to that detected in the invasive tumour cells studied by Weiss *et al.* (1990). Therefore, there is a possibility that both pancreatic trypsinogen and cathepsin B may act independently of each other in the process of cancer invasion and metastasis, like other different classes of proteases involved in the proteolytic modification of the matrix barrier.

It is not clear how the pancreatic trypsinogen peptides are activated, because this protease is thought to be released into the extracellular matrix of carcinoma cells in an inactive form. Activation of this protease is an important regulatory step in the degradation of the extracellular matrix. At least three possible mechanisms can be postulated. These include autoactivation (Colomb *et al.*, 1979), activation by cathepsin B (Greenbaum *et al.*, 1959) and activation by duodenal enterokinase or enteropeptidase refluxed into the pancreatic duct (McCutcheon, 1968; Hadorn *et al.*, 1974) or absorbed into the portal circulation (Talbot *et al.*, 1984). In addition, human pancreatic trypsinogen occurs as two variants differing slightly from one another in biochemical properties such as isoelectric point, susceptibility to inhibitors and substrate specificity (Rinderknecht & Geokas, 1973). Therefore, it will be important to determine which isoform may play a role in tumour invasion and metastasis.

### References

BANSAL, R., AHMAD, H. & KIDWAI, J.R. (1980). *In vitro* conversion of proinuslin to insulin by cathepsin B in isolated islets and its inhibition by cathepsin B antibodies. *Acta Diabetol. Lat.*, **17**, 255–266.

COLOMB, E., FIGARELLA, C. & GUY, O. (1979). The two human trypsinogens: evidence of complex formation with basic trypsin inhibitor. *Biochim. Biophys. Acta*, **570**, 397–405.

CONLEY, C.R., SCHEITHAUER, B.W., WEILAND, L.H. & VAN HEERDEN, J.A. (1987). Diffuse intraductal papillary adenocarcinoma of the pancreas. *Ann. Surg.*, **205**, 246–249.

CUBILLA, A.L. & FITZGERALD, P.J. (1978). Pancreas cancer. *Pathol. Annu.*, **1**, 241–289.

ERDEL, M., TREFZ, G. & SPIESS, E. (1990). Localization of cathepsin B in two human lung cancer cell lines. *J. Histochem. Cytochem.*, **38**, 1313–1321.

GAVANAUGH, P.G., SLOANE, B.F., BAJLOWSKI, A., GARIC, G.J., GASIC, T.B. & HONN, K.V. (1983). Involvement of a cathepsin B-like cysteine protease in platelet aggregation induced by tumor cells and their shed membrane vesicles. *Clin. Exp. Metastasis*, **1**, 297–308.

GREENBAUM, L.M., HIRSHKOWITZ, A. & SHOICHET, I. (1959). The activation of trypsinogen by cathepsin B. *J. Biol. Chem.*, **234**, 2885–2890.

HADORN, B., HESS, J., TROESCH, V., VERHAAGE, W., GOETZE, H. & BENDER, S.W. (1974). Role of bile acids in the activation of trypsinogen by enterokinase: disturbance of trypsinogen activation in patients with intrahepatic biliary atresia. *Gastroenterology*, **66**, 548–555.

HALILA, H., LEHTOVIRTA, P. & STENMAN, U.H. (1988). Tumor-associated trypsin inhibitor (TATI) in ovarian cancer. *Br. J. Cancer*, **57**, 304–307.

HUHTALA, M.L., PERSONEN, K., KALKKINEN, N. & STENMAN, U. (1982). Purification and characterization of a tumor-associated trypsin inhibitor from the urine of a patient with ovarian cancer. *J. Biol. Chem.*, **257**, 13713–13716.

JAPANESE PANCREATIC SOCIETY (1986). *General Rules for Surgery and Pathological Studies on Cancer of the Pancreas*, 3rd edn, Kanehara: Tokyo.

KOIVUNEN, E., HUHTALA, M.L. & STENMAN, U.H. (1989). Human ovarian tumor-associated trypsin. *J. Biol. Chem.*, **264**, 14095–14099.

KOIVUNEN, E., RISTIMAKI, A., ITKONEN, O., OSMAN, S., VUENTO, M. & STENMAN, U.H. (1991*a*). Tumor-associated trypsin participates in cancer cell-mediated degradation of extracellular matrix. *Cancer Res.*, **51**, 2107–2112.

KOIVUNEN, E., SAKSELA, P.O., OSMAN, S., HUHTALA, M.L. & STENMAN, U.H. (1991*b*). Human colon carcinoma, fibrosarcoma and leukemia cell lines produce tumor-associated trypsinogen. *Int. J. Cancer*, **47**, 592–596.

KOPPEL, P., BAICI, A., KEIST, R., MATZKER, S. & KELLER, R. (1984). Cathepsin B-like proteinase as a marker for metastatic tumor cell variants. *Exp. Cell Biol.*, **52**, 293–299.

MCCUTCHEON, A.D. (1968). A fresh approach to the pathogenesis of pancreatitis. *Gut*, **9**, 296–310.

MARKS, W.H., OHLSSON, K. & POLLING, A. (1984). Immunocytochemical distribution of trypsinogen and pancreatic secretory trypsin inhibitor in normal and neoplastic tissues in man. *Scand. J. Gastroenterol.*, **19**, 673–676.

MISZCZUK-JAMSKA, B., MERTEN, M., GUY-CROTTE, O., AMOURIC, M., CLEMENTE, F., SCHOUMACHER, R.A. & FIGARELLA, C. (1991). Characterization of trypsinogens 1 and 2 in two human pancreatic adenocarcinoma cell lines; CFPAC-1 and CAPAN-1. *FEBS Lett.*, **294**, 175–178.

MOROHOSHI, T., KANDA, M., ASANUMA, K. & KLOPPEL, G. (1989). Intraductal papillary neoplasms of the pancreas: a clinicopathologic study of six patients. *Cancer*, **64**, 1329–1335.

MORT, J.S. & RECKLIES, A.D. (1986). Interrelationship of active and latent secreted human cathepsin B precursors. *Biochem. J.*, **233**, 57–63.

PIETRAS, R.J. & ROBERTS, J.A. (1981). Cathepsin B-like enzymes: subcellular distribution and properties in neoplastic and control cells from human ectocervix. *J. Biol. Chem.*, **256**, 8536–8544.

POUR, P.M. (1985). Induction of unusual pancreatic neoplasms, with morphologic similarity to human tumors, and evidence for their ductal/ductular cell origin. *Cancer*, **55**, 2411–2416.

POUR, P.M. (1988). Mechanism of pseudoductular (tubular) formation during pancreatic carcinogenesis in the hamster model: an electron-microscopic and immunohistochemical study. *Am. J. Pathol.*, **130**, 335–344.

RECKLIES, A.D., TILTMAN, K.J., STOKER, T.A.M. & POOLE, A.R. (1980). Secretion of proteinases from malignant and nonmalignant human breast tissue. *Cancer Res.*, **40**, 550–556.

RICKAERT, F., CREMER, M., DEVIERE, J., TAVARES, L., LAMBILLIOTT, J.P., SCHRODER, S., WURBS, D. & KLOPPEL, G. (1991). Intraductal mucin-hypersecreting neoplasms of the pancreas: a clinicopathologic study of eight patients. *Gastroenterology*, **101**, 512–519.

RINDERKNECHT, H. & GEOKAS, M.C. (1973). Anionic and cationic trypsinogens (trypsins) in mammalian pancreas. *Enzyme*, **14**, 116–130.

ROZHIN, J., ROBINSON, D. & STEVENS, M.A. (1987). Properties of a plasma membrane-associated cathepsin B-like cystein proteinase in metastatic B-16 melanoma variants. *Cancer Res.*, **47**, 6620–6628.

SLOANE, B.F., ROZHIN, J., JOHNSON, K., TAYLOR, H., CRISSMAN, J.D. & HONN, K.V. (1986). Cathepsin B: association with plasma membrane in metastatic tumors. *Proc. Natl Acad. Sci. USA*, **83**, 2483–2487.

SLOANE, B.F. (1990). Cathepsin B and cystains: evidence for a role in cancer progression. *Semin. Cancer Biol.*, **1**, 137–152.

STENMAN, U.H., KOIVUNEN, E. & VUENTO, M. (1988). Characterization of a tumor-associated serine protease. *Biol. Chem. Hoppe-Seyler*, **369 (Suppl.)**, 9–14.

TALBOT, R.W., GRANT, D.A.W. & HERMON-TAYLOR, J. (1984). Displacement of endogenous enterokinase into portal venous blood and bile following luminal perfusion of the proximal small intestine in guinea pigs. *Dig. Dis. Sci.*, **29**, 1009–1014.

TRYGGVASON, K., HOYTYA, M. & SALO, T. (1987). Proteolytic degradation of extracellular matrix in tumour invasion. *Biochim. Biophys. Acta*. **907**, 191–217.

WEISS, R.E., LIU, B.C.S., AHLERING, T., DUBEAU, L. & DROLLER, M.J. (1990). Mechanisms of human bladder tumor invasion: role of protease cathepsin. *Br. J. Urol.*, **144**, 798–804.